# Detection of several carbapenems resistant and virulence genes in classical and hyper-virulent strains of *Klebsiella pneumoniae* isolated from hospitalized neonates and adults in Khartoum

**DOI:** 10.1186/s13104-020-05157-4

**Published:** 2020-07-01

**Authors:** Aalaa Mahgoub Albasha, Esraa hassan Osman, Saga Abd-Alhalim, Elianz F. Alshaib, Leena Al-Hassan, Hisham N. Altayb

**Affiliations:** 1 Department of Microbiology, College of Medical Laboratory Science, Sudan University of Science and Technology, Khartoum, Sudan; 2 Department of Global Health and Infection, Brighton and Sussex Centre for Global Health Research, Brighton and Sussex Medical School, Brighton, UK; 3 Biochemistry Department, Faculty of Sciences, King Abdulaziz University, Jeddah, 21452 Saudi Arabia

**Keywords:** *K. pneumoniae*, MDR, XDR, hvKp, Nosocomial infection and, Sudan

## Abstract

**Objective:**

*Klebsiella pneumoniae* (*K. pneumoniae*) involves both community-acquired and nosocomial infections. It is responsible for a wide variety of infections, including infections of the urinary tract, pneumonia, bacteremia, meningitis, wound infection and purulent abscesses. We constructed this study to detect several carbapenems resistant and virulence genes in classical and hyper-virulent strains of *K. pneumoniae* isolated from hospitalized neonates and adults in Khartoum state.

**Results:**

Seventy percent of the isolates were resistant to ceftazidime, 18(30%) to ciprofloxacin, 23(38.3%) to chloramphenicol, 24(40%) to gentamicin and 8% to imipenem, 35% were multidrug-resistant, and 7% extensively drug-resistant, all blood isolates (n = 14) were resistant to ceftazidime. *entB* was the most predominant virulence gene (93.3%), followed by *mrkD* (78.3%), *kfu* (60%), *K2* (51.7%), *magA* (18.3%) and *rmpA* (5%). *bla*_OXA-48_ was the most predominant carbapenem-resistant gene (68.3%), followed by *bla*_NDM_ (10%), *bla*_*KPC*_ (8.3%), and *bla*_IMP_ (3.3%). Eight hyper-virulent strains were positive for *bla*_OXA-48_ and two for *bla*_NDM_ genes.

## Introduction

*Klebsiella pneumoniae (K. pneumoniae)* is a non-motile, capsulated gram-negative rod about 1–2 µm long, and is a facultative anaerobe [1]. It is a common cause of urinary tract, soft-tissue, and central nervous system infections, in addition to endocarditis, and cases of severe bronchopneumonia, sometimes with chronic destructive lesions and multiple abscess formation in the lungs. In many cases, localized infections lead to bacteremia [1].

There are two types of *K. pneumoniae* strains “classic” (cKp), usually non-virulent, and drug-resistant gene producer and usually associated with hospital infections, while the other type is a hypervirulent (hvKp) drug-sensitive strain [2]. *K. pneumoniae* possesses different virulence and antimicrobial resistance genes associated with various clinical conditions [3].

Carbapenemase enzymes of *K. pneumoniae* can resist most β-lactam-ring-containing antibiotics, including carbapenems, and thus conferring resistance to these drugs [4]. Ambler molecular class A *K. pneumoniae* carbapenemase (KPC), class B; Verona integron metallo-betalactamases types (VIM), Imipenemase (IMP) and New Delhi metallo-betalactamase (NDM) and class D oxacillinase-48 (OXA-48) are frequently isolated from severe hospital infections [5].

Carbapenem-resistant hypervirulent strains of *K. pneumonia* are one of the most important organisms that cause fatal nosocomial infections [6]. Recently, increasing reports of resistance to carbapenem in healthcare-associated with *K. pneumonia* infections have been documented in Sudan [7–9]. The mortality rate of carbapenem-resistant *K. pneumoniae* bacteremia could reach 50% of cases [10].

However, to date, there are no published data in Sudan about the distribution and epidemiology of various types of Carbapenemases and virulence genes on hvKp and cKp strains circulating in Khartoum hospitals. This information is of great importance to understand their local epidemiology and to establish eradication and prevention procedures. Thus, this study was conducted to detect and to characterize the common virulence and carbapenem-resistant genes of hvKp and cKp strains isolated from hospitalized patients in different hospitals in Khartoum state.

## Main text

### Methods

A total of 60 isolates of *Klebsiella pneumoniae* were obtained from hospitalized patients (45 adults, and 15 neonates) in different hospitals of Khartoum State, during the period from January 2017 to March 2017. These isolates were collected and identified at hospitals as a part of their routine clinical procedure.

### Bacterial identification

The isolates were re-identified by gram stain, standard biochemical methods (urease test, indole test, and carbohydrates fermentation test, motility test, and citrate utilization test) [11, 12], and by *K*. *pneumoniae* species-specific primers (Table 1) targeting the 16S rRNA gene. Antibiotic susceptibility testing was done by the Kirby Bauer disc diffusion method on Mueller–Hinton agar, the following commonly used antibiotics for the treatment of *K. pneumonia* infection in Sudan were selected; ciprofloxacin (5 mcg), gentamicin (10 mcg), ceftazidime (30 mcg), imipenem (10 mcg), and chloramphenicol (30) (HiMedia Laboratories Pvt. Ltd. Mumbai, India), the results of sensitivity tests were interpreted according to Clinical And Laboratory Standards Institute (CLSI) guidelines [13]. *E. coli* ATCC 25922 and *K. pneumonia* (ATCC 700603) were used as quality control strains.

**Table 1:** Primers sequences and PCR protocols used in this study

Protocols	Temperature cycling	Marker	Sequence (5–3′)	Amplicons size (bp)	References
1st	35 cycles at 94 °C for 30 s, 58 °C for 90 s and 72 °C for 90 s	16 s rRNA	F. ATTTGAAGAGGTTGCAAACGAT R.TTCACTCTGAATTTTCTTGTGTTC	130	[38]
2nd	30 cycles at 94 °C for 30 s, 60 °C for 45 s, and 72 °C for 60 s	*mrkD*	F. AAGCTATCGCTGTACTTCCGGCA R. GGCGTTGGCGCTCAGATAGG	340	^a^ [39]
		*entB*	F. GTCAACTGGGCCTTTGAGCCGTC R. TATGGGCGTAAACGCCGGTGAT	400	
		*rmpA*	F. CATAAGAGTATTGGTTGACAG R. CTTGCATGAGCCATCTTTCA	461	
		*K2*	F. CAACCATGGTGGTCGATTAG R. TGGTAGCCATATCCCTTTGG	531	
		*kfu*	F. GGCCTTTGTCCAGAGCTACG R. GGGTCTGGCGCAGAGTATGC	638	
		*magA*	F. GGTGCTCTTTACATCATTGC R. GCAATGGCCATTTGCGTTAG	1283	
3rd	35 cycles at 94 °C for 20 s, 56 °C for 10 s, 72 °C for 20 s	*NDM*	F. GGTTTGGCGATCTGGTTTTC R. CGGAATGGCTCATCACGATC	521	[39, 40] (Mushi et al. 2014) [17, 37]
		*IMP*	F. TTGACACTCCATTTACAG R. GATTGAGAATTAAGCCACTCT	232	
4th	35 cycles at 94 °C for 45 s, 52 °C for 1 min, and 72 °C for 1 min	*KPC*	F. CATTCAAGGGCTTTCTTGCTGC R. ACGACGGCATAGTCATTTGC	498	
		*OXA*-*48*	F. GCTTGATCGCCCTCGATT R. GATTTGCTCCGTGGCCGAAA	281	

*s* second*, F* Forward*, R *Reverse*, bp *base pair

^a^Annealing time changed from 90 s to 45 s

Capsule stain was used to detect capsule [14]. String test was used to differentiate between hvKp and cKp strains: if the grown colonies of *K. pneumoniae* form a string > 5 mm in length using a sterile loop, this demonstrates the hypermucoviscosity phenotype [15].

### DNA extraction and detection of virulent and resistant genes

DNA was extracted using the guanidine chloride method [16]. The DNA samples were stored at − 80 °C until used for PCR.

A primer sets targeting virulence, and carbapenem-resistant genes of *K*. *pneumoniae* are shown in Table 1. The primers were dissolved according to manufacturer guidelines to prepare 10 pmol/μl in all PCR reactions.

### PCR conditions

PCR was carried out in a 20 μl volume using the Maxime PCR PreMix kit (iNtRON Biotechnology, Seongnam, Korea), 1 μl of each forward and reverse primer (10 pmol/μL), 2 μl of DNA, and then the volume was completed to 20 μl by distilled water. Four multiplex and single reaction PCR protocols were used for amplification of 16S rRNA, resistant and virulence genes, the initial melting temperature for all was 95 °C for 5 min, and a final extension was at 72 °C for 10 min. Details of annealing temperatures are listed in Table 1.

### Statistical analysis

Data of research was analyzed using SPSS. Frequencies and Chi square test was used for comparison of different correlations and associations between variables (*p* value ≤ 0.05).

## Results

### Demographic data

Sixty *K. pneumoniae* isolates were obtained from different hospitals in Khartoum State, 27 (45%) were from females, and 33 (55%) from males, 37 (61.7%) were from urine, 14 (23.3%) were from the blood of neonatal and adult sepsis, 5 (8.3%) were from wound swab, and 4 (6.7%) were from sputum.

### String test

Out of sixty *K. pneumoniae* isolates, 10 (16.7%) were hypermucoviscous, and 50 (83.3%) isolates were classic.

### Susceptibility test results

Most strains, 42 (70%), were resistant to ceftazidime, 18 (30%) to ciprofloxacin, 23 (38.3%) to chloramphenicol, 24 (40%) to gentamicin and only 5 (8%) resistant to imipenem. Multidrug resistant isolates were detected in 12 of urine isolates, 7 of blood, and 2 of wound swab isolates. Three neonatal blood isolates and one adult wound swab were showed extensively drug-resistant, more results are shown in Table 2.

**Table 2:** Susceptibility testing profile of *K. pneumoniae* strains among different clinical specimens and age groups

	Ciprofloxacin				Chloramphenicol				Gentamicin				Imipenem				Ceftazidime			
	Sensitive		Resistant		Sensitive		Resistant		Sensitive		Resistant		Sensitive		Resistant		Sensitive		Resistant	
*Sex N = 60*																				
Male	20	(48%)	13	(72%)	17	(46%)	16	(70%)	17	(47%)	16	(67%)	31	(56%)	2	(40%)	10	(56%)	23	(55%)
Female	22	(52%)	5	(28%)	20	(54%)	7	(30%)	19	(53%)	8	(33%)	24	(44%)	3	(60%)	8	(44%)	19	(45%)
p	0.082				0.076				0.143				0.49				0.95			
*Sample N = 60*																				
Urine	27	(64%)	10	(56%)	24	(65%)	13	(57%)	24	(67%)	13	(54%)	37	(67%)	0	(0%)	15	(83%)	22	(52%)
Blood	8	(19%)	6	(33%)	7	(19%)	7	(30%)	6	(17%)	8	(33%)	10	(18%)	4	(80%)	0	(0%)	14	(33%)
Wound swab	3	(7%)	2	(11%)	3	(8%)	2	(9%)	3	(8%)	2	(8%)	4	(7%)	1	(20%)	1	(6%)	4	(10%)
Sputum	4	(10%)	0	(0%)	3	(8%)	1	(4%)	3	(8%)	1	(4%)	4	(7%)	0	(0%)	2	(11%)	2	(5%)
*p*	0.80				0.95				0.83				0.23				0.38			
Total	42		18		37		23		36		24		55		5		18		42	

*p *= *p*-value, N = number

### PCR results

#### Detection of *K. pneumoniae* carbapenem-resistant and virulence genes

At least one of carbapenem-resistant genes were detected in 76.7% (46/60) of isolates; 68.3% (41/60) were positive for *bla*_OXA-48_ gene, 10% (6/60) were positive for *bla*_*NDM*_ gene, 8.4% (5/60) were positive for *bla*_*KPC*_ gene, and 3.3% (2/60) were positive for *bla*_*IMP*_ gene. One neonatal blood isolate possesses three carbapenem-resistant genes (*bla*_*KPC*_, *bla*_OXA-48_, and *bla*_*IMP*_), six isolates possess two genes (four possess *bla*_OXA-48_ and *bla*_*NDM*_, two possess *bla*_OXA-48_ and *bla*_*KPC*_), and thirty-nine isolates possess one gene (34 *bla*_OXA-48_, two *bla*_*NDM*_*, two bla*_*KPC*_, and *one bla*_*IMP*_) and the remaining (14) were negative for all carbapenem-resistant genes. Eight hyper-virulent strains were harboring *bla*_OXA-48_ and two harboring *bla*_NDM_ genes.

For virulence genes *mrk*D detected in 47 (78.3%) isolates, *ent*B in 56 (93.3%), *rmp*A in 3(5%), *K2* in 31 (51.7%), *kfu* in 36 (60%) and *mag*A in 8 (13.3%) isolates.

There was no significant statistical association between the presence of virulence genes and carbapenems resistant genes except between *ent*B and NDM (*p*-value = 0.005) (Table 3). A total of 92% (43/47) of *mrk*D gene-positive isolates were positive for one or more carbapenem-resistant genes. There was a strongly significant association between the presence of *mrkD* and *entb* genes (*p*-value = 0.0005), they were co-existed in 46 isolates.

**Table 3:** The association between *K. pneumoniae* virulence and carbapenems resistant genes production

	*IMP*				*OXA*-*48*				*KPC*				*NDM*			
	Positive		Negative		Positive		Negative		Positive		Negative		Positive		Negative	
*mrkD*																
Positive	1	(2)	46	(98%)	32	(68%)	15	(32%)	4	(9%)	43	(91%)	4	(9%)	43	(91%)
Negative	1	(8%)	12	(92%)	9	(69%)	4	(31%)	1	(8%)	12	(92%)	2	(15%)	11	(85%)
*p*	0.33				0.93				0.92				0.47			
*entB*																
Positive	2	(4%)	54	(96%)	39	(70%)	17	(30%)	5	(9%)	51	(91%)	4	(7%)	52	(93%)
Negative	0	(0%)	4	(100%)	2	(50%)	2	(50%)	0	(0%)	4	(100%)	2	(50%)	2	(50%)
*p*	0.70				0.42				0.51				0.005			
*rmpA*																
Positive	0	(0%)	3	(100%)	1	(33%)	2	(67%)	0	(0%)	3	(100%)	0	(0%)	3	(100%)
Negative	2	(4%)	55	(96%)	40	(70%)	17	(30%)	5	(9%)	52	(91%)	6	(11%)	51	(89%)
*p*	0.74				0.18				0.59				0.51			
*k2*																
Positive	1	(3%)	30	(97%)	21	(68%)	10	(32%)	1	(3%)	30	(97%)	3	(10%)	28	(90%)
Negative	1	(3%)	28	(97%)	20	(69%)	9	(31%)	4	(14%)	25	(86%)	3	(10%)	26	(90%)
*p*	0.94				0.92				0.14				0.93			
*kfu*																
Positive	0	(0%)	36	(100%)	26	(72%)	10	(28%)	1	(3%)	35	(97%)	3	(8%)	33	(92%)
Negative	2	(8%)	22	(92%)	15	(63%)	9	(38%)	4	(17%)	20	(83%)	3	(13%)	21	(88%)
*p*	0.08				0.43				0.05				0.60			
*magA*																
Positive	1	(13%)	7	(88%)	7	(88%)	1	(13%)	1	(13%)	7	(88%)	1	(13%)	7	(88%)
Negative	1	(2%)	51	(98%)	34	(65%)	18	(35%)	4	(8%)	48	(92%)	5	(10%)	47	(90%)
*p*	0.12				0.21				0.65				0.80			

## Discussion

In this study, eight hyper-virulent strains of *K. pneumoniae* were reported positive for carbapenems resistant genes (*OXA*-*48* and *NDM)*. The presence of these strains in the clinical setting will complicate clinical practice and will cause fatal nosocomial infections [6]. Although antimicrobial-resistant hvKP strains are rarely reported worldwide [17–19], here in Sudan, they appear to be more prevalent.

Eight neonatal blood isolates were multidrug-resistant, and three of them were extensively resistant to all antibiotics that were used. Consequently, the emergence of MDR pathogens would increase the mortality and morbidity and prolong hospitalization and cost of treatment [20].

All neonatal blood isolates (12) were resistant to ceftazidime. Ceftazidime-resistant *Klebsiella pneumoniae* (CRKP) in the pediatric oncology units of some Sudanese hospitals may be the cause of recent reports of high mortality rate associated with *K. pneumoniae* infections among this group in different Sudanese hospitals [21]. According to Schiappa [22], high resistance rates to ceftazidime could be due to the presence of a predominant enzyme (TEM-10) responsible for ceftazidime resistance in bloodstream isolates.

The isolates showed varying degrees of resistance to the other antibiotics; ciprofloxacin 30%, gentamicin 40%, and ceftazidime (70%). Resistance to these antibiotics may also be due to the presence of Extended-Spectrum Beta-lactamases (ESBLs) and other mechanisms like efflux pumps and porin mutations [23], which were not covered in this study.

Although chloramphenicol is used as a treatment of choice for MDR gram-negative bacilli bacteria [24], 38% of our isolates were resistant to it, which may be caused by transferable enzymatic resistance to aminoglycosides, that is common in some hospitals [25].

In the current study, 94% (51/54) of the isolates harboring carbapenem-resistant genes were phenotypically susceptible to imipenem. This confirms what Walsh [26] said that this gene is not stable and relies upon other synergistic mechanisms to mediate resistance against carbapenems. In addition to imipenem, other antibiotics were analyzed in this study. Although five strains of *K. pneumoniae* in this study were resistant to imipenem, only three of them were positive for carbapenem-resistant genes (*OXA*-*48*), the rest two strains may possess other carbapenem-resistant genes not covered in this study or possessed another mechanism of resistance [27].

Of 46 *K. pneumoniae* isolates detected of having carbapenem-resistant genes, 10 had multiple genes co-occurring. This finding agrees with Ali & Omer [28] and Satir [29], which showed a multiplicity of genes in their isolates.

A total of 80% (4/5) of *KPC* and 100% (2/2) of *IMP* genes were positive among infant blood samples, and this may be due to organisms harboring these genes having a high ability to cause systemic infections, particularly in immunocompromised patients [30].

In this study, we found the essential gene for *K. pneumoniae* siderophores system *entB* gene is positive in 93.3% of all *K. pneumoniae* isolates, the rest (6.7%) of isolates that do not possess *entB* may contain other enterobactin (*ent*A, C, D, E or F), or other siderophores systems like yersiniabactin or aerobactin as reported by Lawlor [20]. Furthermore, *mrk*D gene is presented in 78.3% of the isolate. This gene has been found to be important in adhesion, as reported by Chen et al. (2012) [30]. The *rmp*A gene was detected in 5% of isolates, in contrast with Aljanaby and Alhasani [20], who found the *rmp*A gene present in 62.5% of *K. pneumoniae* isolates. This difference may be attributed to its mode of inheritance as plasmid-mediated, as mentioned by [20], indicating the limited spread of this gene in our local strains in Sudan.

The capsular serotype gene *K2* was present in 51.7% of isolates; the rest of isolates may contain other capsular serotypes, as mentioned by Ho [31]. This study showed that *K*2 is present in 80% of hypermucoviscous strains, indicating that there is a relationship between the presence of *K*2 gene and hypermucoviscous strains of *K. pneumoniae*, which is in agreement with the study by Guo [32] which found that *K*2 is the most common capsular serotype in hypermucoviscous strain. In contrast to other studies [20, 33–35], which found *K1* was the most prevalent capsular serotype among hypermucoviscous *K. pneumoniae*.

The *kfu* gene (which codes for an iron uptake system) was present in 60% of isolates. The study showed no association between the presence of *kfu* gene and hypermucoviscosity. This finding disagrees with previous studies [20, 36, 37], which showed that *kfu* gene is associated with hypermucoviscosity phenotype, which may be attributed to diversity in geographical locations of studies.

The *mag*A gene was found in 13.3% of isolates. The study showed no association between the presence of *magA* gene and hypermucoviscous strains. Although this gene is highly essential for *K. pneumoniae,* which confirms bacterial mucoviscosity, its prevalence among local isolates is not high, suggesting that other genes play a role in the formation of mucoviscosity [20].

## Conclusion

The study reported for the first time in Sudan the following findings:

1. Presence of carbapenems resistant genes in hyper-virulent strains of *K. pneumoniae* isolated from hospitalized patients.
2. Presence of MDR and XDR strains of *K. pneumoniae* in neonatal ward in some Sudanese hospitals.

## Limitations

- Low sample size.
- DNA sequencing not done due to financial issues.

## Data Availability

The datasets used and analyzed during the current study are available at 10.6084/m9.figshare.12401684.

## Abbreviation

*bla*: β-lactamase
cKp: Classic *K. pneumoniae*
CLSI: Clinical and Laboratory Standards Institute
CPS: Capsular polysaccharide
*ent*B: Enterobactin B
ESBL: Extended-spectrum β- lactamase
*hvKp*: Hyper-virulent *Klebsiella pneumoniae*
IPM: Imipenem
*kfu*: Klebsiella Ferric Uptake
KPC: *Klebsiella pneumoniae* carbapenemase
OXA-48: Oxacillinase 48
*mag*A: Mucoviscosity-Associated Gene A
MDR: Multi Drug Resistant
*mrk*D: Mannose Resistant Klebsiella like hemoagglutinin D
NDM: New Delhi metallo
PCR: Polymerase chain reaction
*rmp*A: Regulatory of Mucoid Phenotype A
SPSS: Statistical Package for the Social Sciences
XDR: Extensively drug-resistant
